# Comparison of Nursing Workload Between Direct Anterior Approach Versus Posterolateral Approach for Hip Arthroplasty in Femoral Neck Fractures: A Prospective Cohort Study

**DOI:** 10.1002/nop2.70805

**Published:** 2026-11-03

**Authors:** Pingping Xu, Fengli Gao, Huiyue Zhang, Baojing Zhao, Kai Yang, Jingqian Huang, Lu Chen, Yongwei Pan, Han Cao

**Affiliations:** ^1^ Beijing Tsinghua Changgung Hospital, School of Clinical Medicine Tsinghua University Beijing China; ^2^ Medical Data Science Center, Beijing Tsinghua Changgung Hospital, School of Clinical Medicine, Tsinghua Medicine Tsinghua University Beijing China

**Keywords:** femoral head replacement, femoral neck fractures, hip arthroplasty, nurse care, nurse workload

## Abstract

**Aims:**

To compare the perioperative nursing workload between the direct anterior approach and the posterolateral approach for hip arthroplasty in femoral neck fractures.

**Design:**

A single‐centre, prospective observational cohort study.

**Methods:**

The study population consisted of 27 orthopaedic nurses who provided perioperative care for patients who underwent a direct anterior approach or posterolateral approach. Perceived nursing workload was assessed using the NASA Task Load Index, while actual workload was evaluated using the Orthopaedic Nurse Work Responsibilities Scale, which quantified the cumulative time (in minutes) required to complete nursing tasks. The unit of analysis was each nurse–patient interaction, with assessments conducted at five time points: preoperative Day 1, postoperative Days 1–3 and discharge day. Data were analysed using linear mixed‐effects models with random intercepts for both nurse and patient to account for repeated measurements and clustering.

**Results:**

Twenty‐seven orthopaedic nurses providing perioperative care for 65 patients completed the study. The two patient groups were comparable in baseline characteristics. The actual nursing workload was similar between the two groups. For perceived workload, the main effect of surgical approach was not statistically significant (*β* = −0.59, 95% CI [−2.042, 0.862], *p* = 0.421). However, the group‐by‐time interaction was significant, and the perceived workload was significantly higher for nurses caring for PLA patients on postoperative Day 3 (*β* = −1.364, 95% CI [−2.267, −0.461], Bonferroni‐corrected *p* = 0.003) and at discharge (*β* = −1.406, 95% CI [−2.309, −0.503], Bonferroni‐corrected *p* = 0.002). Descriptive analyses suggested that nurses at higher competency levels tended to report lower perceived and actual workload than their less experienced counterparts, though this was not formally tested.

**Conclusion:**

Surgical approach was not associated with actual nursing workload but appeared to be associated with higher perceived workload later in the recovery phase, which may reflect institution‐specific care pathways rather than the surgical approach alone. Nurse experience was inversely related to both perceived and actual workload. These hypothesis‐generating findings suggest that surgical approach and nurse experience may merit consideration when planning staffing and training to optimize workload and enhance patient care.

**Implications for Nursing Practice:**

The findings suggest that nursing staffing and training programs could consider both the surgical approach and nurse experience levels to optimize workload management, support nurse well‐being and enhance the quality of perioperative patient care. Nurse managers could consider providing additional support to patients receiving PLA when they first get out of bed and upon discharge to alleviate the perceived workload of the nurse.

**Patient or Public Contribution:**

Patients and members of the public were not involved in the design, conduct, reporting or dissemination plans of this research. They participated solely as study participants, providing data through workload assessments during their perioperative care. We sincerely thank both the patients and nurses for their participation in this study.

**Trial Registration:**

This study was registered on the Chinese Clinical Trial Registry (ChiCTR2500100878)

## 1 Introduction

The incidence of femoral neck fractures (FNFs) among the elderly is rising globally, becoming a leading cause of mortality, disability and economic burden in ageing populations, with 10 million people expected to be disabled by the disease each year (Sing et al. 2023). According to the results of a recent cohort study, for these patients, early hip arthroplasty (HA) could improve prognosis by reducing the duration of time spent in a deleterious state of bleeding, inflammation, pain and immobility (HIP ATTACK Investigators 2020). However, the optimal surgical approach for HA remains controversial (Chalmers et al. 2023; Peuchot et al. 2024). The traditional posterolateral approach (PLA) has been widely used and proven over time to be associated with shorter operative time (Rykov et al. 2017), less intraoperative bleeding (Wang et al. 2024) and a significantly reduced risk of the procedure, especially for the elderly and senior citizens, a population that is also prone to femoral neck fractures. While the direct anterior approach (DAA), gaining prominence in recent decades, offers potential advantages including a lower dislocation rate, enhanced pain relief, faster postoperative mobilization and superior short‐term functional recovery as measured by patient‐reported outcomes at 2–4 weeks (Jin, Yao, et al. 2023; Jin, Wang, et al. 2023; Lu et al. 2025; van der Sijp et al. 2018). Some studies report comparable outcomes between PLA and DAA in terms of hospitalization duration and long‐term recovery (Jin, Yao, et al. 2023; Jin, Wang, et al. 2023), yet disparities in postoperative care demands, particularly concerning dislocation risk and pain management, have not been thoroughly investigated.

For clinical nurses, these differences may translate into varying workloads in postoperative care. Preliminary observations indicate that PLA patients often require more intensive monitoring and guidance to mitigate dislocation risk, potentially increasing nursing burden. Workload, encompassing both objective tasks and subjective perceptions, critically influences care quality. Research in intensive care settings demonstrates that excessive workload correlates with missed nursing interventions (Rasooly et al. 2021; Tubbs‐Cooley et al. 2019), heightened stress and adverse patient outcomes, including higher rates of falls and medication errors (Carlesi et al. 2017; Jin, Yao, et al. 2023; Jin, Wang, et al. 2023). Despite these risks, no studies have systematically examined whether HA surgical approaches differentially impact nursing workload, a gap that may hinder optimal resource allocation and patient safety. This prospective observational cohort study is the first to evaluate nursing workload disparities between PLA and DAA from the caregivers' perspective. By quantifying differences in postoperative care workload and demands, our findings will inform targeted staffing protocols, enhance nurse well‐being and ultimately improve patient recovery outcomes for hip arthroplasty.

## 2 Methods

### 2.1 Design

We conducted a prospective observational cohort study to assess the differences in nursing tasks and workload between DAA and PLA for patients undergoing total hip arthroplasty and bipolar hemiarthroplasty. All study procedures were performed in accordance with the guidelines outlined in the Declaration of Helsinki and approved by the Ethics Committee of Beijing Tsinghua Changgung Hospital (Approval No. 24717–4‐02, approved on February 11, 2025). Written informed consent was obtained from all participating patients and nurses prior to enrollment. All data were de‐identified to protect participant confidentiality.

### 2.2 Perioperative Nursing Protocols for the DAA and PLA Groups

The perioperative care for patients undergoing hip arthroplasty, regardless of whether the DAA or the PLA approach is used, is essentially the same. This includes regular monitoring of vital signs, administering prescribed antibiotics, analgesia and treatment to prevent deep vein thrombosis in the lower extremities; regular turning to prevent pressure sores; and regular changing of hospital gowns and bed linens to ensure patient hygiene and comfort. Preoperatively, patients and their families receive necessary education, such as explaining the surgical procedure, providing dietary guidance and instructing them on proper bedpan and bowel techniques. Emotional support is also provided to alleviate patient anxiety. Postoperatively, patients receive guidance on activity, self‐management education and preparation for discharge (Sun et al. 2024).

The difference in nursing care between the two approaches lies in postoperative guidance and assistance with patient activity. Patients using the PLA approach are required to use positioning aids such as T‐pillows to maintain proper hip alignment, avoid excessive flexion or adduction of the hip joint and use riser chairs and toilet seats to maintain hip angle (Di Martino et al. 2024). For patients using the direct anterior approach (DAA), the main focus is on instructing them to avoid sitting cross‐legged without requiring other positional restrictions.

### 2.3 Sampling and Recruitment

We enrolled patients with FNF admitted to our ward between February 11, 2025 and July 31, 2025, who required hemiarthroplasty or THA via DAA or PLA. In our ward, both approaches are used, with the choice of approach based on the physician's preference and training and were not influenced by the research team. We selected consecutive patients who underwent surgery within a specific timeframe for our study, provided, naturally, that they fulfilled the following inclusion and exclusion criteria. The inclusion criteria were adult patients (≥ 60 years) with femoral neck fractures who underwent DAA or PLA hip arthroplasty. The exclusion criteria were patients with cognitive impairments, cerebrovascular sequelae that affected walking, severe pulmonary complications, lower extremity deep vein thrombosis or pathological fractures. These conditions were excluded because they are known to substantially increase nursing care demands, such as enhanced supervision, mobility assistance, respiratory support and anticoagulation monitoring, which could confound the workload differences attributable to the surgical approach itself.

Since no previous study has directly compared the nursing workload between the DAA method and the PLA method, it is not possible to perform a formal a priori sample size calculation based on an effect size specific to this comparison. For reference, we utilized the most relevant existing data from a previous study (Pretto et al. 2009), which reported total nursing workloads of 1667 ± 42 min and 1721 ± 88 min for two groups of hip replacement patients in an orthopaedic ward and a general ward, respectively. Assuming a test power of 80% and a two‐sided *α* value of 0.05, this calculation indicates that at least 26 patients are required per group. However, since the cited study did not specifically compare the DAA and PLA approaches, this estimate may not accurately reflect the true effect size in our repeated‐measures design. Consequently, all eligible patients admitted during the 6‐month recruitment period were included in the study (30 patients in the DAA group and 35 patients in the PLA group), with the final sample size determined by feasibility rather than statistical power.

The nurses included in the study were registered orthopaedic nurses who were directly involved in the perioperative care of patients. They were classified into four clinical competency levels: Nurse Level 1 (N1, novice), Nurse Level 2 (N2, competent), Nurse Level 3 (N3, proficient/specialist) and Nurse Level 4 (N4, expert) (Yang et al. 2023). Nurses who were not directly involved in patient care, nurses who were on short‐term rotation or internships or nurses who withdrew during the study period were excluded.

### 2.4 Data Collection

Patient basic information and disease‐related data were obtained from medical records. This information was compiled in a pre‐established Excel spreadsheet. The nursing staff's perceived workload (measured by the NASA Task Load Index [Tubbs‐Cooley et al. 2018]) and actual workload (measured by the Orthopaedic Nurse Work Responsibilities scale [Olthof et al. 2018]) were assessed using paper‐based forms on the following days: preoperative Day 1 (PRD1), postoperative Days 1, 2 and 3 (POD1, POD2, POD3) and on the day of discharge. The unit of analysis was each nurse–patient interaction. At each observation time point, each patient received care from only one nurse; however, the nurses providing care to each patient varied across different time points. After each participating nurse completed care for an enrolled patient (PRD1, POD1, POD2, POD3 and discharge day), the nurse assessed both their subjective perception of workload and their actual workload. A total of 325 interaction records were completed, with no missing data.

The NASA Task Load Index (NASA‐TLX) was administered in its Chinese version, translated and validated by Liang (Liang et al. 2019) and used with the author's permission. The scale comprises six items: mental demand, physical demand, temporal demand, performance, effort and frustration. Each item was rated on a 21‐point scale ranging from 0 (low) to 20 (high); for the performance item, a rating of 0 indicated “perfect” and 20 indicated “failure.” The overall mental workload score was calculated using the raw TLX method by summing the raw scores of the six items and dividing by 6, yielding a score ranging from 0 to 20, with higher scores indicating greater perceived workload. In the present study, the internal consistency of the NASA‐TLX was acceptable, with a Cronbach's α of 0.707.

The Orthopaedic Nurse Work Responsibilities scale (Table 1) was adapted for use by orthopaedic nursing staff based on the “time‐and‐motion” methodological framework described by Olthof et al. (2018). In their study, Olthof et al. used direct observation to record all activities performed by orthopaedic nursing staff and categorized these activities into six domains: medical technical tasks, communication, patient transfer, feeding, activities of daily living and other activities. Drawing upon this classification framework, we adapted it to the context of our orthopaedic nursing practice, categorizing nursing tasks into five domains: patient communication, nursing procedures, patient transfer, activity guidance and feeding assistance. For each task, the time required (in minutes) was recorded and served as its score. The total scale score was the sum of all task scores. Thus, a higher total score directly reflects a greater time investment and, consequently, a heavier actual workload. The internal consistency of the scale in the present study was acceptable, with a Cronbach's α of 0.776.

**TABLE 1:** Orthopaedic nurse work responsibilities scale.

Nursing type	Work responsibilities
Communication with patients	Communicate with patients and their families
	Provide emotional support
	Explain precautions and orthopaedic treatments to patients and families
Nursing procedures	Measure important patient parameters (blood pressure, temperature, pulse, etc.)
	Measure fluid intake and output
	Accompany doctors during rounds
	Record patient condition
	Prepare medications and administer them
	Skin and wound care
	Catheter care
	Intravenous therapy
Transfer of patients	Transport to radiology for examination
	Transport to other departments
Activity guidance	Assist patients in getting out of bed to use the toilet or bedside commode
	Change patient gowns and bed sheets
	Assist with bed activities (including turning, sitting up, etc.)
Dietary assistance	Provide food and water, assist patients with eating

### 2.5 Data Analyses

The data analysis was conducted using SPSS version 27.0 (IBM Corp., Armonk, NY, USA). Descriptive statistics were used to summarize the demographic and clinical characteristics of the patients, including age, gender and type of surgical approach. Continuous variables were presented as mean ± standard deviation (SD), while categorical variables were expressed as frequencies and percentages.

To compare the perceived workload and actual workload between the DAA and PLA groups across multiple follow‐up time points, linear mixed‐effects models were employed. In the models, surgical approach (DAA vs. PLA), time (follow‐up time points) and the group‐by‐time interaction were entered as fixed effects. The PLA group served as the reference category for surgical approach, and preoperative Day 1 (PRD1) served as the reference time point. Random intercepts were included for both nurse ID and patient ID to account for the correlations among repeated measurements nested within the same nurse and the same patient. The restricted maximum likelihood (REML) method was used for parameter estimation. *Post hoc* pairwise comparisons were performed using the Bonferroni correction to adjust for multiple comparisons. Separate linear mixed‐effects models were constructed for actual workload and perceived workload, respectively. All statistical tests were two‐tailed, and a *p*‐value of less than 0.05 was considered statistically significant. The surgical approach was decided through discussion between the surgeon and the patient rather than by randomization; potential confounders such as procedure type, patient characteristics, nurse experience, staffing ratios, operative time, blood loss and mobilization timing could not be adjusted for. The potential influence of these factors was acknowledged in the Limitations section.

## 3 Results

### 3.1 Demographic Data

#### 3.1.1 Patients

During the study period, a total of 73 patients with femoral neck fractures underwent hip arthroplasty, and 65 patients were included in this study based on the inclusion and exclusion criteria. Figure 1 illustrates the patient enrollment process. Thirty patients underwent DAA, while 35 patients underwent PLA. The basic information was shown in Table 2. Baseline characteristics revealed no significant differences between groups (*p* > 0.05), indicating comparability of the study cohorts. It is worth noting that there were significant differences between the two groups in terms of intraoperative blood loss (*p <* 0.001) and operative time (*p <* 0.001), reflecting inherent differences between the two surgical methods rather than baseline imbalances.

**FIGURE 1:**
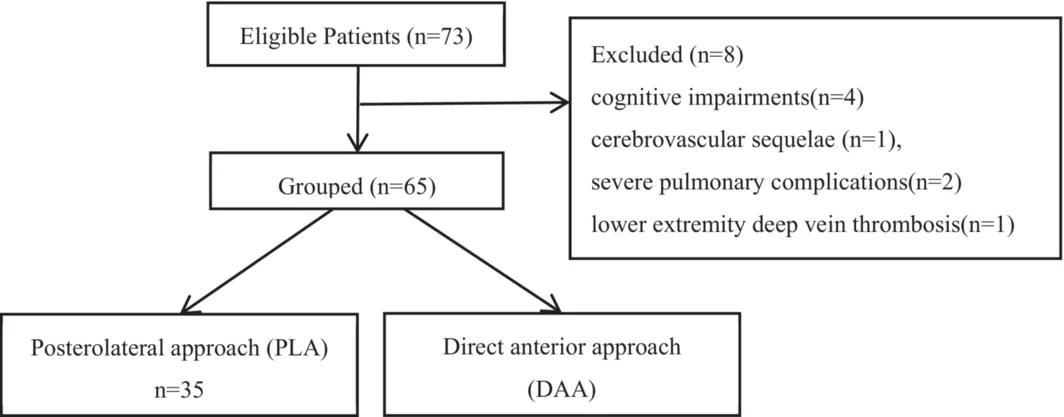
Patient enrollment process. DAA, direct anterior approach; PLA, posterolateral approach.

**TABLE 2:** Socio‐demographic profile of the patients (*n* = 65).

Variables	DAA (*n* = 30)	PLA (*n* = 35)	*Z/χ* ^*2*^	*p*
	M (P_25_, P_75_)/*n* (%)			
Patient gender			0.484	0.487
Female	19 (43.2%)	25 (56.8%)		
Male	11 (52.4%)	10 (47.6%)		
Patient age (years)	74 (69, 81)	77 (67, 84)	−0.020	0.984
Number of diagnoses	4 (2, 7)	5 (3, 7)	−0.457	0.647
Length of hospital stay (days)	9.5 (8, 12.5)	10 (8, 15)	−0.344	0731
Affected side			0.001	0.969
Left side	13 (46.4%)	15 (53.6%)		
Right side	17 (45.9%)	20 (54.1%)		
Surgical procedure			0.973	0.324
Bipolar hemiarthroplasty	26 (49.1%)	27 (50.9%)		
Total Hip Arthroplasty	4 (33.3%)	8 (66.7%)		
Intraoperative blood loss (mL)	200 (50, 200)	50 (30, 75)	−3.821	< 0.001*
Duration of surgery (min)	120 (104, 134)	89 (69, 107.5)	−4.074	< 0.001*

Abbreviations: DAA, direct anterior approach; PLA, posterolateral approach.

* *p* < 0.05.

#### 3.1.2 Nurses

Table 3 summarizes the socio‐demographic characteristics of the nurses, including 1 male and 26 females, with ages ranging from 21 to 35 years. Nurses were classified into nurse Level 1 (N1), nurse Level 2 (N2) and nurse Level 3 (N3) based on their clinical experience and expertise. Although the hospital‐wide system comprises five tiers (N0–N4), this study was conducted in a unit where direct patient care is provided exclusively by nurses in these three core tiers. Therefore, the study cohort consisted of 27 nurses, N1 (*n* = 9), N2 (*n* = 10) and N3 (*n* = 8), who were responsible for the daily care of all 65 patients.

**TABLE 3:** General information characteristics of nurses (*n* = 27).

Variables	M (P25, P75)/*n* (%)
Age	31 (24, 33)
Gender	
Male	1 (3.7%)
Female	26 (96.3%)
Grade	
N1	9 (33.3%)
N2	10 (37.0%)
N3	8 (29.7%)

Abbreviations: N1, nurse Level 1; N2, nurse Level 2; N3, nurse Level 3.

### 3.2 Nursing Workload

The results of the linear mixed‐effects models are presented in Table 4. With regard to perceived workload, the main effect of surgical approach was not statistically significant (*β* = −0.59, 95% CI [−2.042, 0.862], Bonferroni‐corrected *p* = 0.421). A statistically significant group difference was confirmed between the DAA and PLA groups on postoperative day 3 and discharge day regarding perceived workload (*β* = −1.364, 95% CI [−2.267, −0.461], Bonferroni‐corrected *p* = 0.003, *β* = −1.406, 95% CI [−2.309, −0.503], Bonferroni‐corrected *p* = 0.002). For actual workload, neither the main effect of surgical approach (Bonferroni‐corrected *p* = 0.543) nor the group‐by‐time interaction (*p* > 0.05) reached statistical significance, indicating that actual workload was comparable between the two surgical approaches across all time points.

**TABLE 4:** Results of linear mixed‐effects models for nursing workload (*n* = 65).

Outcomes	DAA (*n* = 30) *M ± SD*	PLA (*n* = 35) *M ± SD*	*β*	*t*	95% CI	*p*
Actual nursing workload	205.41 ± 137.49	215.43 ± 109.12	−13.99	−0.61	−59.22, 31.24	0.543
PRD1	189.93 ± 129.29	201.49 ± 94.04	—	—	—	—
POD1	231.13 ± 146.30	240.09 ± 129.61	3.777	0.122	−57.270, 64.822	0.903
POD2	217.47 ± 133.39	230.09 ± 129.64	−2.767	−0.089	−63.813, 58.280	0.929
POD3	208.43 ± 137.86	217.20 ± 119.25	0.724	0.023	−60.322, 61.770	0.981
Discharge day	178.70 ± 126.55	184.86 ± 101.64	−0.100	−0.003	−61.145, 60.945	0.997
Perceived nursing workload	10.31 ± 3.43	11.54 ± 2.92	−0.59	−0.808	−2.042, 0.862	0.421
PRD1	11.68 ± 3.45	12.26 ± 2.31	—	—	—	—
POD1	12.04 ± 3.09	12.67 ± 2.67	−0.587	−0.128	−0.961, 0.843	0.898
POD2	10.05 ± 3.03	10.75 ± 2.69	−0.906	−0.198	−0.993, 0.812	0.844
POD3	10.97 ± 3.25	12.9 ± 2.91	−1.364	−2.98	−2.267, −0.461	0.003*
Discharge day	7.89 ± 3.26	9.87 ± 2.89	−1.406	−3.07	−2.309, −0.503	0.002*

Abbreviations: DAA, direct anterior approach; PLA, posterolateral approach; POD, postoperative day; PRD, preoperative day.

* *p* < 0.05.

A line chart analysis was used to compare trends across nurse levels. The results revealed distinct differences in both actual and perceived workload among the tiers. Notably, a negative correlation was observed between nurse level and workload measures. Specifically, nurses at higher levels (N3) reported significantly lower levels of perceived task load and also demonstrated lower actual workload scores compared to their N2 and N1 counterparts. The detailed results are presented in Figure 2 and Figure 3.

**FIGURE 2:**
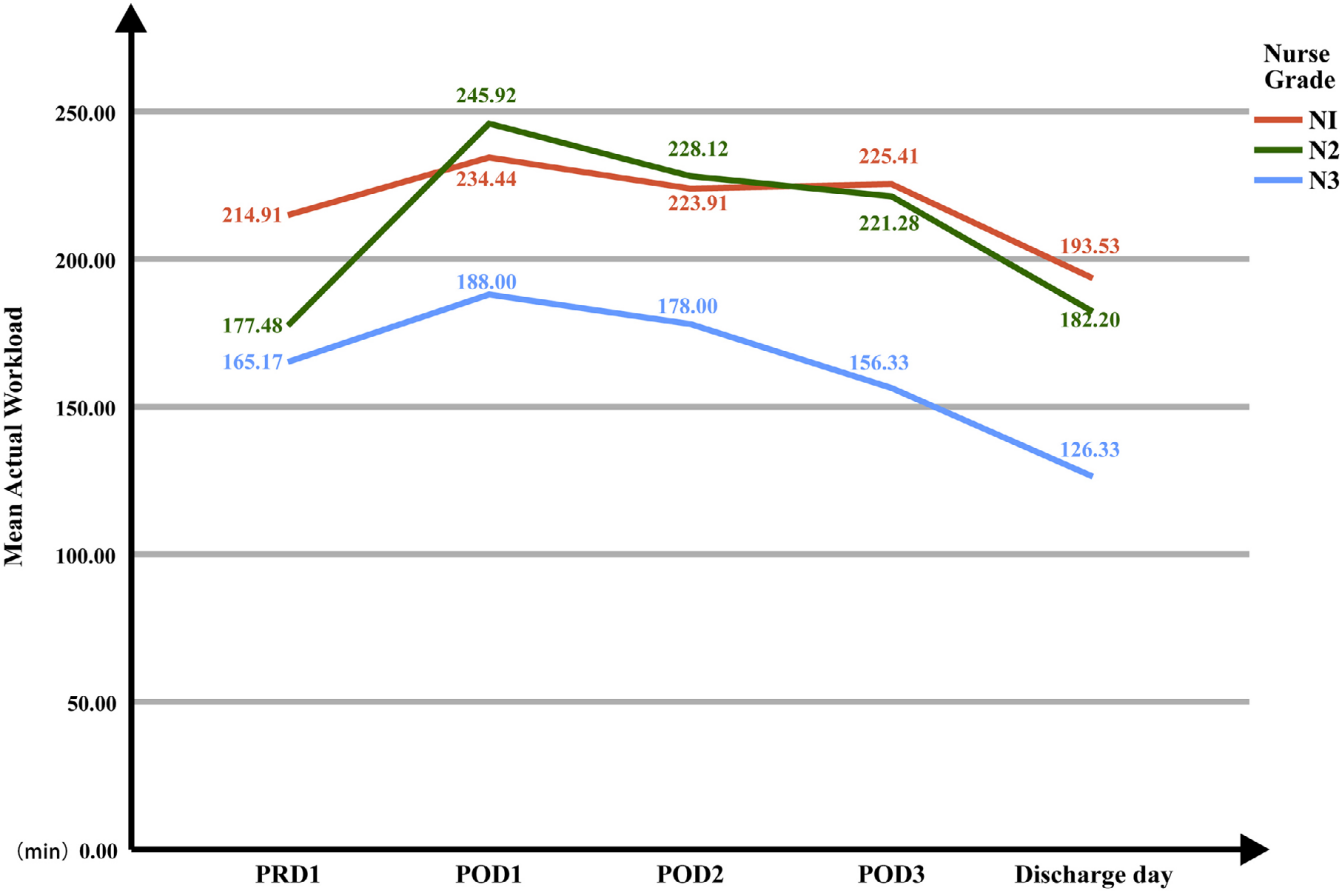
Mean Actual Workload by Nurse Grade. N1, nurse Level 1; N2, nurse Level 2; N3, nurse Level 3; PRD, pre‐operative day; POD, post‐operative day.

**FIGURE 3:**
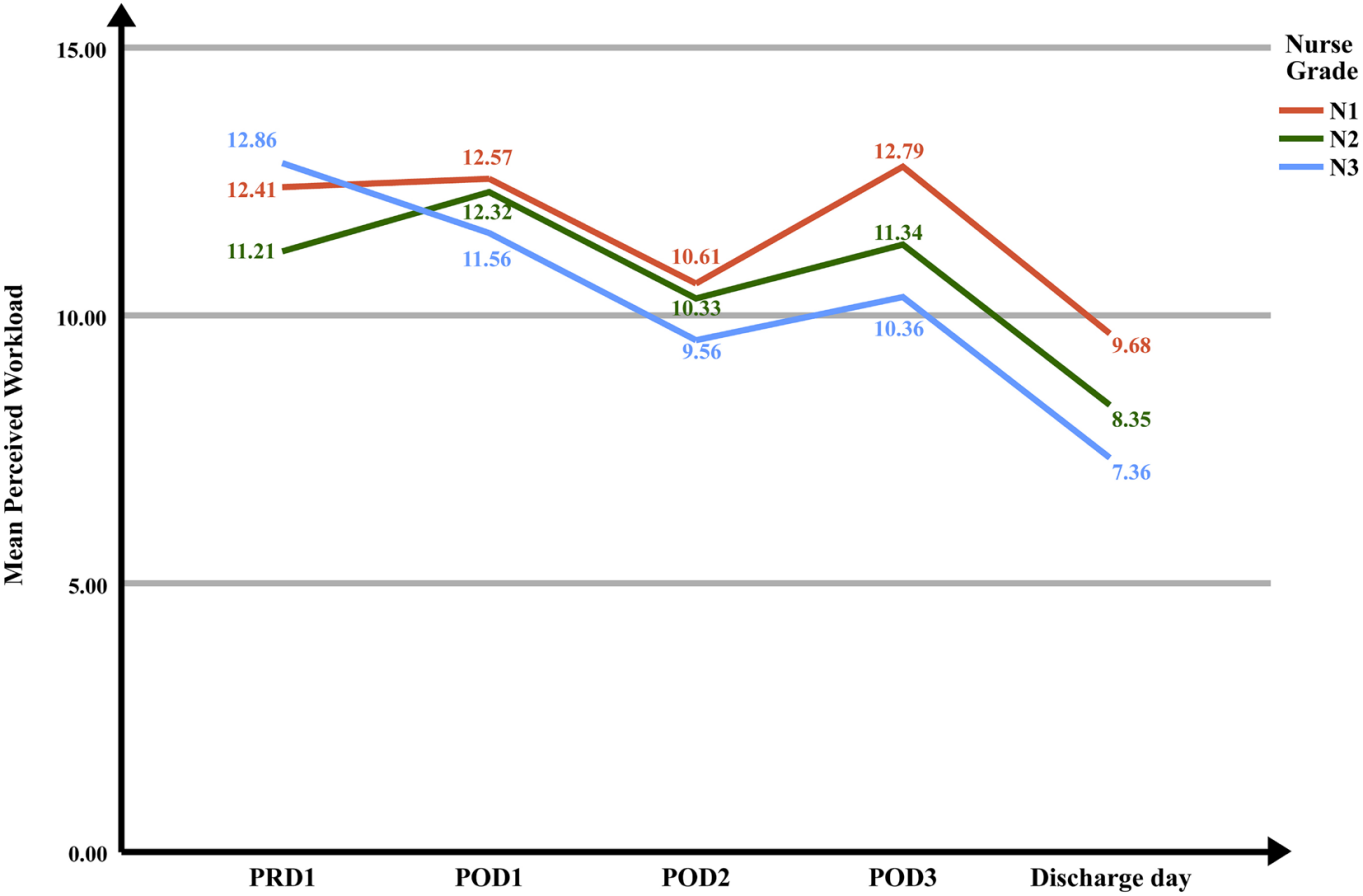
Mean Perceived Workload by Nurse Grade. N1, nurse Level 1; N2, nurse Level 2; N3, nurse Level 3; PRD1, pre‐operative Day 1; POD1, post‐operative Day 1; POD2, post‐operative Day 2; POD3, post‐operative Day 3.

## 4 Discussion

Nursing workload directly impacts the quality and actual level of care that nurses provide to patients (Alghamdi 2016). Understanding nursing workload is a key component of any process aimed at measuring nursing intensity and achieving optimal nursing practice. However, the number of nurses required to provide optimal care, often reflected in nurse‐to‐patient ratios, does not truly reflect the actual or perceived workload of nurses. For patients who underwent PLA, the risks of falls and joint dislocation during their first mobilization attempt on postoperative day three may contribute to the nursing staff's perceived workload. This concern among nurses persists even when patients are discharged and transition to a home environment.

According to our nursing care protocol, DAA patients are encouraged to begin ambulation, assisted by a walker, as early as the first postoperative day (Yang et al. 2021), owing to the minimal soft tissue disruption and better early stability associated with this approach (Yin et al. 2024). In contrast, PLA patients typically undergo their first mobilization on the third postoperative day under direct supervision from both physicians and nursing staff, which may have contributed to the increased perceived workload reported by nurses in the PLA group on POD3 (Skowronek et al. 2025). The first mobilization attempt on postoperative day three presents multiple challenges that elevate nursing burden in the PLA group. At this stage, patients remain at considerable risk for falls (Wu et al. 2025) and hip dislocation (Neyisci et al. 2020), events that require close monitoring and heightened vigilance from nursing staff to ensure patient safety (Skowronek et al. 2025; Ulivi et al. 2021). Given that such complications represent serious adverse events in orthopaedic care, particularly among elderly patients, the increased demand for supervision and support naturally leads to a higher perception of workload (Ulivi et al. 2021). Furthermore, the discharge process itself imposed additional perceived workload on the PLA group. Patients discharged after PLA often required more comprehensive education regarding hip precautions, assistive device use and home mobility management due to the later and more cautious mobilization schedule. This increased need for structured instruction and reassurance before discharge further heightened nurses' perceived workload on discharge day.

The absence of a statistically significant difference in actual nursing workload between the DAA and PLA groups is a noteworthy finding. While measurement data indicate that the total time spent on direct patient care activities, such as vital signs monitoring, medication administration and wound care, was comparable between the two groups, nurses consistently reported higher perceived workload in the PLA group, particularly surrounding the first mobilization event and discharge. This discrepancy underscores a fundamental limitation in conventional nursing workload metrics, which often prioritize countable tasks and time allocation while overlooking the qualitative intensity of care (Alghamdi 2016; Li et al. 2025). Specifically, although the first leave bed attempt on POD3 and the pre‐discharge education for PLA patients did not substantially increase the total recorded task time, these high‐risk clinical junctures demanded more intense attention, risk anticipation and decision‐making responsibility from nursing staff. This invisible burden, including sustained vigilance, anxiety over potential serious complications and heightened communication responsibilities, constitutes a core component of perceived nursing workload, yet it remains largely invisible within conventional time‐and‐task models (Hoogendoorn et al. 2021; Olthof et al. 2018). In contrast, while nursing care for DAA patients also required time investment, the inherent technical advantages of this approach, associated with lower complication risks and a smoother recovery trajectory, resulted in relatively lower cognitive and emotional tension for nurses during task execution, thereby contributing to a correspondingly reduced perceived workload.

Our study also explored the influence of nursing experience on both perceived and actual workload. Descriptive analyses suggested a trend whereby senior nurses (N3) reported lower workload measures compared to intermediate (N2) and junior nurses (N1) across both perceived and actual workload dimensions. However, because nurse competency level was not formally tested in the mixed‐effects models, these observations should be regarded as exploratory and hypothesis‐generating rather than confirmatory. Although exploratory, this pattern is consistent with existing literature suggesting that greater nursing experience may mitigate the cognitive and emotional burdens associated with demanding clinical tasks, thereby lowering perceived workload (Ivziku et al. 2022; Lee et al. 2022; Liu et al. 2024; Wang et al. 2021; Wong 2024). Experienced nurses often possess greater proficiency in patient monitoring, early complication recognition and task prioritization, all of which may contribute to streamlined workflows and reduced redundant tasks (Liu et al. 2024). However, these proposed mechanisms remain speculative in the context of our study, and formal testing of the nurse experience effect is warranted in future research. Although these findings are exploratory, they tentatively suggest that nursing management may benefit from structuring shifts and patient assignments to blend expertise across seniority levels, thereby distributing cognitive load more effectively and creating opportunities for mentorship. However, given the descriptive nature of the nurse‐level findings, such recommendations should be considered preliminary and require confirmation in future studies.

### 4.1 Implications for Nursing Practice

The results of this study have important implications for clinical practice. First, it is essential to recognize that differences in postoperative care protocols, such as timing of mobilization, can lead to significant variations in perceived nursing workload. Nurse managers could consider adjusting nurse–patient ratios or providing additional support during high‐risk days, particularly for patients undergoing procedures that involve delayed mobilization, such as PLA. This can help alleviate the burden on nursing staff, particularly in settings where falls and dislocations are significant concerns.

Moreover, the negative correlation between nurse experience and workload perception suggests that providing opportunities for professional development and mentorship could play a supportive role in improving nurse efficiency and reducing stress. Investing in continuing education programmes focused on specialized care for elderly patients, as well as strategies to manage common postoperative complications, can further enhance nursing performance and job satisfaction. This approach not only benefits nurses but also contributes to better patient outcomes by ensuring that care is delivered in a timely and effective manner.

### 4.2 Limitations and Future Research

While this study provides valuable insights into the impact of surgical approach and nurse experience on nursing workload, limitations must be acknowledged. First, this was a single‐centre, prospective, observational cohort study. The results may reflect the organizational structure, staffing patterns, and clinical workflow specific to our department rather than being solely attributable to the surgical approach itself. Second, the sample size was determined by feasibility rather than a formal power calculation and was relatively small, which limited statistical power and precluded subgroup analyses. Third, our inclusion criteria were deliberately strict to minimize confounding, excluding patients with cognitive impairment, cerebrovascular sequelae or severe pulmonary complications. This resulted in a narrow patient population and limited representativeness, thereby restricting generalizability. Fourth, the perceived workload questionnaire used in this study, while previously applied in other studies, has not been extensively validated as a gold‐standard instrument, and further validation is needed. Fifth, because the choice of surgical approach was determined jointly by the surgeon and the patient rather than through random assignment, we were unable to adjust for potential confounding factors, including surgical procedure type, patient characteristics, nurse experience, nurse‐to‐patient ratios, operative time, intraoperative blood loss and mobilization timing, and their influence on the observed results cannot be ruled out. Finally, due to the observational nature of the study design, our findings can only suggest associations and cannot establish causality. Future multi‐centre studies with larger and more diverse patient populations are needed to confirm these preliminary observations and to examine whether the observed differences in workload persist across different hospital settings.

## 5 Conclusion

In this study, the surgical approach was not associated with actual workload but was associated with higher perceived workload later in the recovery phase, which may reflect institution‐specific mobilization and postoperative care pathways rather than the surgical approach alone. This discrepancy highlights a limitation of standard workload metrics, which fail to account for the qualitative burden, including cognitive load and supervisory anxiety, associated with different patient pathways. Descriptive analyses suggested that greater nurse experience tended to be associated with lower perceived and actual workload, although this requires formal testing in future studies. These preliminary findings suggest that tailoring staffing and training to specific surgical approaches and nurse expertise may merit further evaluation. Given the single‐centre design, larger multi‐centre studies are needed to confirm these preliminary observations.

## Author Contributions

**Pingping Xu:** conceptualization, methodology, data curation, investigation, supervision, validation, writing – original draft, writing – review and editing, formal analysis. **Fengli Gao:** supervision, conceptualization, methodology, project administration, writing – review and editing, writing – original draft, resources. **Huiyue Zhang:** conceptualization, methodology, software, data curation, investigation, validation, formal analysis, writing – original draft, writing – review and editing. **Baojing Zhao:** data curation, investigation. **Kai Yang:** investigation, resources. **Jingqian Huang:** data curation, investigation. **Lu Chen:** formal analysis, writing – original draft. **Yongwei Pan:** data curation, methodology, supervision, writing – review and editing. **Han Cao:** data curation, formal analysis, writing – review and editing.

## Funding

The authors have nothing to report.

## Disclosure

No generative artificial intelligence tools were used in the conception, design, conduct, analysis or writing of this study. We have used the Chinese version of the NASA Task Load Index. Permission for its use in this study has been obtained from the Chinese version translator.

## Ethics Statement

All study procedures were performed in accordance with the guidelines outlined in the Declaration of Helsinki and approved by the Ethics Committee of Beijing Tsinghua Changgung Hospital (No. 24717–4‐02).

## Consent

Written informed consent was obtained from all participants or their legal guardians before enrolment.

## Conflicts of Interest

The authors declare no conflicts of interest.

## Supporting information




**Data S1:** STROBE statement.

## Data Availability

The data that support the findings of this study are available on request from the corresponding author. The data are not publicly available due to privacy or ethical restrictions. The STROBE statement for this cohort study is provided as Supporting Information.
